# A new species of snailfish of the genus *Paraliparis* (Liparidae) from the western North Pacific, with a redescription of the poorly known species *Paraliparis mandibularis*

**DOI:** 10.3897/zookeys.968.56057

**Published:** 2020-09-16

**Authors:** Yoshiaki Kai, Kenta Murasaki, Ryo Misawa, Atsushi Fukui, Eisuke Morikawa, Yoji Narimatsu

**Affiliations:** 1 Maizuru Fisheries Research Station, Field Science Education and Research Center, Kyoto University, Nagahama, Maizuru, Kyoto 625-0086, Japan; 2 Institute of Oceanic Research and Development, Tokai University, 3-20-1 Orido Shimizu, Shizuoka 424-8610, Japan; 3 Tohoku National Fisheries Research Institute, Japan Fisheries Research and Education Agency, 25-259 Same, Hachinohe, Aomori, 031-0841, Japan; 4 School of Marine Science and Technology, Tokai University, 3-20-1 Orido, Shimizu, Shizuoka 424-8610, Japan

**Keywords:** Japan, *Paraliparis cephalus*, *Paraliparis flammeus* sp. nov., *Paraliparis mento*, taxonomy, Tohoku

## Abstract

A new snailfish, *Paraliparis flammeus*, is described on the basis of 18 specimens collected off the Pacific coast of Tohoku District, northern Japan at depths of 422–890 m. The new species is distinguished from 28 species of *Paraliparis* described from the North Pacific by the following combination of characters: mouth oblique; uppermost pectoral-fin base below horizontal through posterior margin of maxillary; 60–63 vertebrae, 54–58 dorsal-fin rays, 50 or 51 anal-fin rays, six principal caudal-fin rays, and 17–20 pectoral-fin rays. A maximum likelihood tree based on 106 COI gene sequences (492 bp) of *Paraliparis* recovered a monophyletic group comprising *P. flammeus*, *Paraliparis cephalus*, and *Paraliparis dipterus*. *Paraliparis cephalus* is similar to *P. flammeus* in having an oblique mouth, but it has four caudal-fin rays (vs six rays) and the uppermost pectoral-fin base above a horizontal through the maxillary posterior margin. *Paraliparis dipterus* differs from *P. flammeus* in having a horizontal mouth, 12–14 pectoral-fin rays, and lacking pyloric caeca (present in *P. flammeus*). *Paraliparis flammeus* is most similar to the eastern North Pacific *Paraliparis mento* in having an oblique mouth and the uppermost pectoral-fin base below a horizontal through the posterior margin of the maxillary. However, *P. flammeus* differs from *P. mento* in having six caudal-fin rays (vs five rays) and greater preanal length (29.9–35.3% SL vs 26.7–28.5% SL). A poorly known species, *Paraliparis mandibularis*, previously known from only two specimens collected from Tosa Bay, southern Japan, is redescribed based on the holotype and seven newly collected specimens. It is also similar to the new species but has 27–30 pectoral-fin rays and a shorter pectoral-fin lower lobe (13.8–15.9% SL in *P. mandibularis* vs 16.7–23.4% SL in *P. flammeus*).

## Introduction

Members of the family Liparidae (snailfishes), comprising over 430 species in ca 30 genera, exhibit great diversity in morphology, as well as in geographic and habitat range (Chernova et al. 2004; Nelson et al. 2016; Orr et al. 2019), and they occur worldwide in warm-temperate to cold water habitats ranging from the intertidal to depths exceeding 8,000 m (Nelson et al. 2016; Gerringer et al. 2017). *Paraliparis* Collett, 1879 and related genera have been variously synonymized in previous studies. Although Kido (1988) combined 11 genera under *Paraliparis* following a phylogenetic analysis based on osteological characters, later authors, e.g., Mecklenburg et al. (2002), Chernova et al. (2004), and Nakabo and Kai (2013), considered six of the included genera, e.g., *Elassodiscus* Gilbert & Burke, 1912, *Rhinoliparis* Gilbert, 1896, and *Lipariscus* Gilbert, 1915, as valid. In a recent molecular phylogenetic study, Orr et al. (2019) confirmed that *Paraliparis* was paraphyletic, requiring further taxonomic revision. Nevertheless, present members of the genus are generally distinguishable by the following characters: single nostril; one suprabranchial pore, six branchiostegal rays, more than two rays in the lower lobe of the pectoral fin, and the absence of a pelvic disk, a pseudobranch, a coronal pore and a barbel or a skin flap on the head (Stein et al. 2001; Stein 2012; Murasaki et al. 2018). In its present concept, *Paraliparis* is one of the most speciose genera of the family with ca 140 species, mostly known from depths greater than 200 m (Chernova et al. 2004; Murasaki et al. 2019a, b).

Along the Pacific coast of Tohoku District, northern Honshu Island, Japan, continuous surveys for resource assessments of ground fishes by the Tohoku National Fisheries Research Institute, Japan Fisheries Research and Education Agency, have resulted in the discovery of several new species (Shinohara et al. 2009), including the recent collection of 18 specimens of a previously unknown snailfish of the genus *Paraliparis*. Most closely resembling *Paraliparis mento* Gilbert, 1892 and *Paraliparis mandibularis* Kido, 1985, the specimens have an oblique mouth and a pectoral fin below a horizontal through the posterior maxillary margin but are clearly distinguishable from the latter two species in other morphological characters, as well as DNA barcoding sequence data. They are accordingly described herein as members of a new species. *Paraliparis mandibularis*, a rare species previously known only from the holotype and one non-type specimen (Kido 1988), is redescribed here in detail on the basis of the holotype and seven newly collected specimens from southern Japan.

## Materials and methods

Methods for counts and measurements follow Baldwin and Orr (2010), with the descriptive terminology of Stein et al. (2001). Counts of median-fin rays and vertebrae were taken from radiographs. Cephalic pores were observed by staining with Aniline Blue (Wako Chemicals). Selected specimens were cleared and double stained (C&S) for bone and cartilage examination following the protocol of Kawamura and Hosoya (1991), and using an incident-light fluorescence unit (OptoCode; LED 470 MS-EPI) and stereomicroscope. Osteological characters of the holotype were examined by an industrial x-ray and a computed tomography scanning system (Nikon Corporation), with data visualized by VGStudio Max 3.1 (Volume Garaphics GmbH). The specimens examined in this study are deposited in the fish collections of the Faculty of Science and Technology, Kochi University, Japan (**BSKU**); Kyoto University, Kyoto and Maizuru, Japan (**FAKU**); the Marine Science Museum, Tokai University, Shizuoka, Japan (**MSM**); the Smithsonian Institution, National Museum of Natural History, Suitland, USA (**USNM**); and the Burke Museum, University of Washington, Seattle, USA (**UW**).

For DNA barcoding, we attempted to include all the available sequences of congeneric or related species (see Orr et al. 2019) for the robust phylogenetic inference. Total DNA of the present new species and the following species, *Paraliparis atramentatus* Gilbert & Burke, 1912, *Paraliparis cephalus* Gilbert, 1892, *Paraliparis dipterus* Kido, 1988, *Paraliparis hokuto* Murasaki, Takami & Fukui, 2019a, *Paraliparis mento*, *Paraliparis ruficometes* Murasaki, Takami & Fukui, 2018, *Paraliparis variabilidens* Murasaki, Takami & Fukui, 2019b, *Rhinoliparis barbulifer* Gilbert, 1896, was extracted from fin clips preserved in 99.5% ethanol, using the Wizard Genomic DNA Purification Kit (Promega Inc.). The partial Cytochrome Oxidase subunit I (COI) gene was amplified using the primers designed by Folmer et al. (1994) (LCO1490: 5′- GGT CAA CAA ATC ATA AAG ATA TTG G -3′; HCO2198: 5′- TAA ACT TCA GGG TGA CCA AAA AAT CA -3′). The PCR proceeded for 30 cycles, with denaturation at 94 °C for 15 sec, annealing at 45 °C for 15 sec, and extension at 72 °C for 30 sec, using the KAPA2G Robust PCR Kit (KAPA Biosystems). After the PCR products were purified using ExoSAP-IT Express (ThermoFisher Scientific), they were sequenced on an automated DNA sequencer (ABI Prism 310 Genetic Analyzer; ThermoFisher Scientific) using amplification primers and the BigDye Terminator v. 1.1 Cycle Sequencing Kit (ThermoFisher Scientific). All sequences determined here are available from INSDC (International Nucleotide Sequence Database Collaboration) under accession numbers LC556300–LC556314. Together with previously determined sequences of species of *Paraliparis* and the “Paracareprocta” clade of Orr et al. (2019), as well as an outgroup taxon (*Nectoliparis pelagicus* Gilbert & Burke, 1912: see Orr et al. 2019) available from INSDC and BOLD (Barcode of Life Data System), the present sequences were aligned using MAFFT v. 7 (Katoh and Standley 2013). From the aligned sequences, the uncorrected *p*-distance among specimens was calculated with MEGA X (Kumar et al. 2018). In addition, in order to reconstruct a maximum likelihood (ML) tree, the best evolutionary model was found by MEGA X, the Tamura and Nei (1993) model with gamma shape parameter and invariant sites being selected. Branch support was measured using nonparametric bootstrapping with 1,000 replications, based on the same algorithm (Felsenstein 1985).

## Taxonomy

### Paraliparis flammeus

Taxon classificationAnimaliaScorpaeniformesLiparidae

Kai, Murasaki & Fukui sp. nov.

959D81A6-281B-5D9D-87D5-A6CD1D0C07AD

http://zoobank.org/6CC26DDA-647B-492D-93E9-0B13213A3623

**Figure 1.**
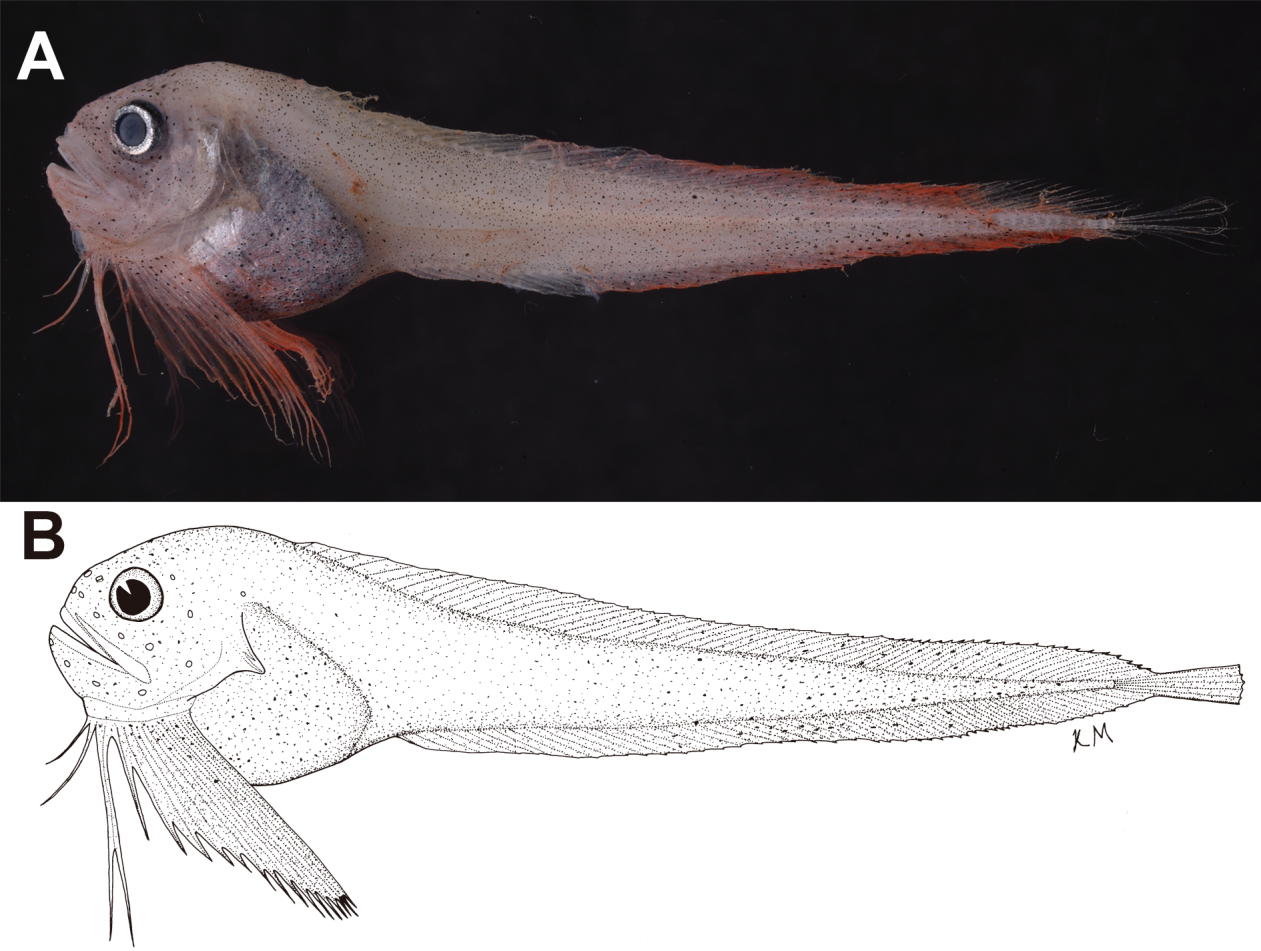
*Paraliparis flammeus* sp. nov., MSM-20-52, holotype, 78.5 mm SL **A** fresh specimen **B** line drawing.

**Figure 2.**
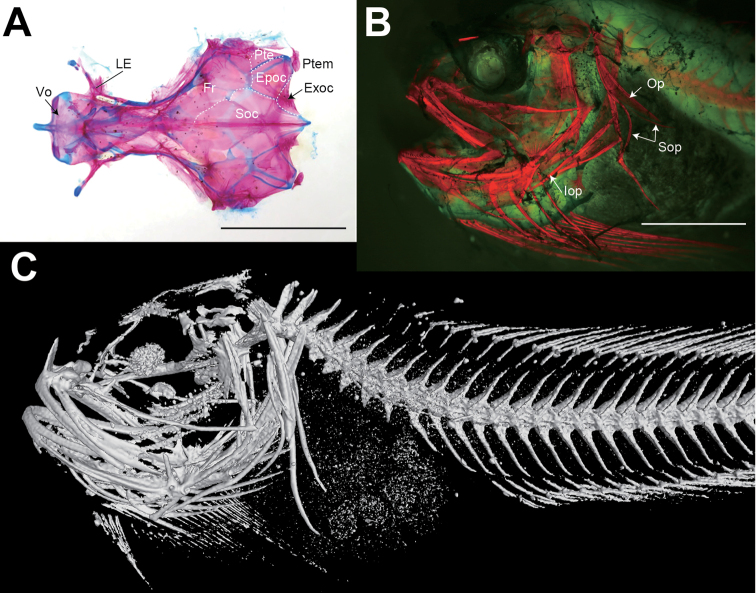
Skeleton of *Paraliparis flammeus* sp. nov. **A, B** C&S specimens, FAKU 147158, paratypes **A** dorsal view of cranium of 68.2 mm SL specimen **B** lateral view of head of 61.2 mm SL specimen **C** CT-scanned holotype, MSM-20-52, holotype, 78.5 mm SL. Abbreviations: Epoc, epiotic; Exoc, exoccipital; Fr, frontal; Iop, interopercle; LE, lateral ethmoid; Op, opercle; Sop, subopercle; Pte, pterotic; Ptem, posttemporal; Soc, supraoccipital; Shp, sphenotic; Vo, vomer. Scale bars: 5 mm (**A, B**).

**Figure 3.**
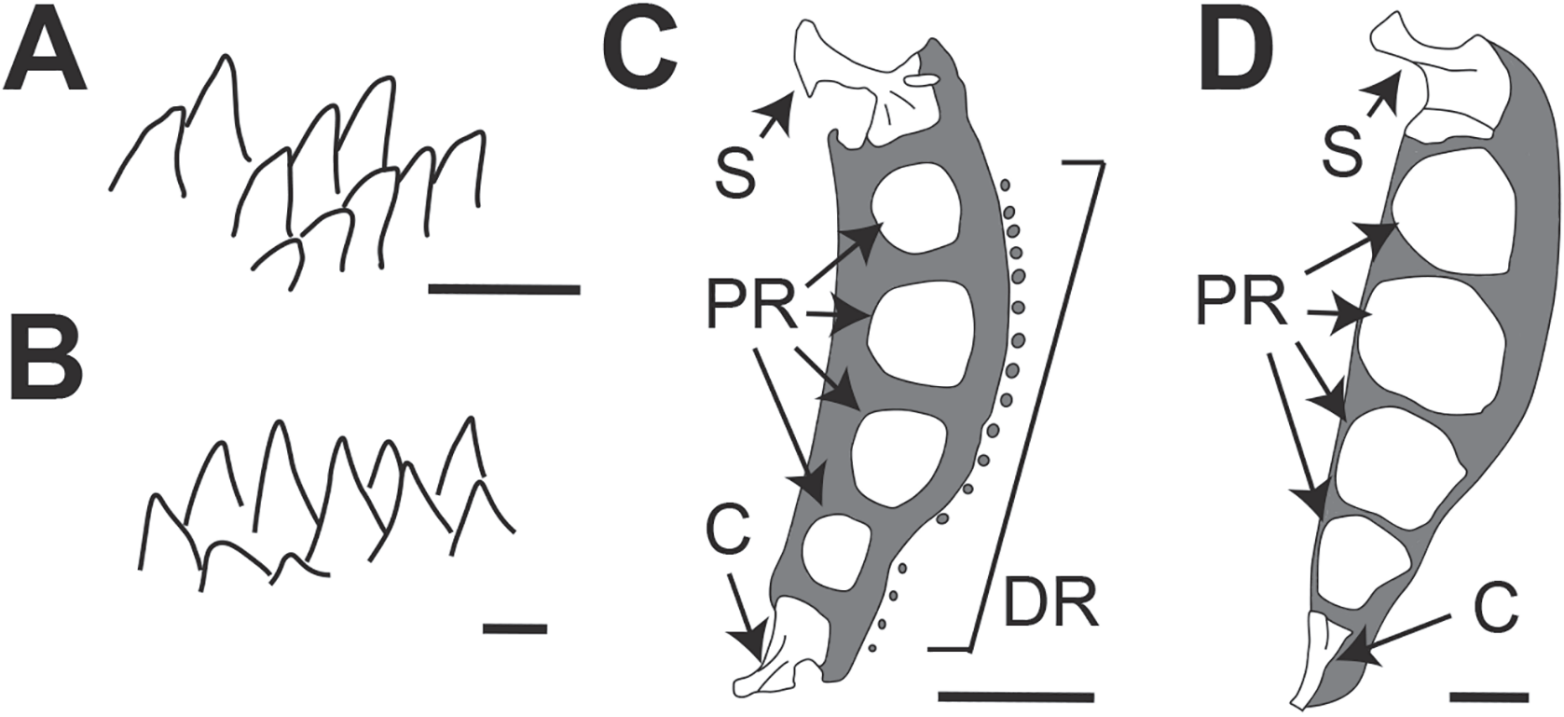
**A, B** teeth on lower jaw **C, D** pectoral girdle **A, C***Paraliparis flammeus* sp. nov., FAKU 147158, paratype, 61.2 mm SL **B, D***P. mandibularis*, BSKU 44286, 110.0+ mm SL. Abbreviations: S, scapula; PR, proximal radials; C, coracoid; DR, distal radials. Scale bars: 0.5 mm (**A, B**); 5 mm (**C, D**).

Figs 1 , 2 , 3A, C [New Japanese name: Homuradama]

#### Holotype.

MSM-20-52, 75.8 mm SL, female, 36.850°N, 141.496°E, 510 m depth, 30 Oct. 2018, coll. K. Murasaki, R/V *Wakataka-maru*, otter trawl.

#### Paratypes.

All specimens were collected by R/V *Wakataka-maru*, otter trawl. FAKU 147147 (INSDC accession: LC556311), 80.4 mm SL, 36.972°N, 141.635°E, 561 m depth, 17 Nov. 2019, coll. R. Misawa; FAKU 147148 (LC556312), 62.1 mm SL, 36.859°N, 141.479°E, 459 m depth, 16 Nov. 2019, coll. R. Misawa; FAKU 147158 (3 specimens, C&S), 49.3–68.2 mm SL, 36.972°N, 141.635°E, 561 m depth, 17 Nov. 2019, coll. R. Misawa; FAKU 147159 (LC556313), 51.1 mm SL, 36.510°N, 141.064°E, 460 m depth, 12 Nov. 2019, coll. R. Misawa; FAKU 147161, 51.5 mm SL, 37.664°N, 141.984°E, 482 m depth, 19 Nov. 2019, coll. R. Misawa; FAKU 147163, 66.4 mm SL, FAKU 147164, 64.4 mm SL, 36.858°N, 141.505°E, 510 m depth, 16 Nov. 2019, coll. R. Misawa; FAKU 147168, 79.7 mm SL, 36.818°N, 141.669°E, 890 m depth, 9 Nov. 2019, coll. R. Misawa; FAKU 147177, 42.5 mm SL, FAKU 147178, 62.1 mm SL, 36.873°N, 141.468°E, 422 m depth, 16 Nov. 2019, coll. R. Misawa; FAKU 147432, 67.3 mm SL, 36.858°N, 141.505°E, 510 m depth, 16 Nov. 2019, coll. R. Misawa MSM-20-53, 64.8 mm SL, male, MSM-20-54, 60.3 mm SL, male, 38.399°N, 142.121°E, 552 m depth, 21 Oct. 2018, coll. K. Murasaki; MSM-20-55, 43.4 mm SL, male, 36.511°N, 141.087°E, 507 m depth, 16 Nov. 2018, coll. K. Murasaki; MSM-20-56, 45.4 mm SL, female, 36.848°N, 141.479°E, 484 m depth, 15 Nov. 2017, coll. K. Murasaki.

#### Diagnosis.

*Paraliparis flammeus* is distinguished from other species of *Paraliparis* by the following combination of characters: mouth oblique; uppermost pectoral-fin base below a horizontal through posterior margin of maxillary; 60–63 vertebrae, 54–58 dorsal-fin rays, 50 or 51 anal-fin rays, 6 principal caudal-fin rays, and 17–20 pectoral-fin rays; pectoral radials 4, moderately large and located medially.

#### Description.

Measurements are shown in Table 1. Paratype data are given in parentheses if different from the holotype.

***Body*** compressed, elongate, deepest at nape, strongly taping posteriorly (Fig. 1). Skin thin, fragile. Head compressed, dorsal profile strongly sloping from nape to snout. Snout deep, blunt, its length almost equal to orbit diameter; not projecting anterior to upper jaw. Mouth oblique, lower jaw slightly protruding beyond (or almost same length as) upper jaw; premaxillary tooth plates matching mandibular tooth plates; maxilla extending to posterior margin of orbit; oral cleft extending to middle of orbit (Figs 1, 2). Premaxillary teeth simple, in 7 (3–8) oblique rows; diastema narrow between premaxillae. Mandibular teeth simple, in 6 (3–7) oblique rows; inner teeth larger; diastema absent at lower jaw symphysis (Fig. 3A). Orbit of moderate size, rounded. Nostril single, with slightly raised rim, at level of mid-orbit. Cephalic sensory pores small (damaged): nasal pores 2, maxillary pores 6, preoperculomandibular pores ≥ 6 (skin damaged in holotype and all paratypes), suprabranchial pore 1; cephalic pore pattern 2-6-6?-1. Chin pores paired, openings well separated on skin surface. Coronal pore absent. Gill slit small, entirely above pectoral fin (or extending ventrally to level of 1 or 2 uppermost pectoral-fin rays), upper margin level with mid-orbit (or between center and ventral rim of orbit). Gill rakers 6–10 (status in holotype unknown), blunt and minute. Tip of opercular flap sharp, angled slightly dorsally, level with ventral rim of orbit (or with posterior margin of maxillary).

***Dorsal-fin*** rays 56 (54–58); anteriormost ray above tip of opercle, posteriormost ray attached membranously to dorsalmost caudal-fin ray. Anteriormost dorsal-fin pterygiophore inserted between neural spines 3 and 4 (2 and 3, or 3 and 4), bearing a single ray. Anal-fin rays 51 (48–51); posteriormost ray attached membranously to ventralmost caudal-fin ray. Vertebrae 63 (60–63), comprising precaudal 9 and caudal 54 (51–54). Pleural ribs absent. Hypurals and parhypural fused into single plate. Caudal fin slender, posterior margin slightly rounded (or truncate). Principal caudal-fin rays 6, dorsal principal rays 3, ventral principal rays 3, no procurrent rays. Pyloric caeca 7 (4–6), short and finger-like, on left side of visceral cavity. Anus below posterior margin of preopercle (or midway between posterior margin of preopercle and posterior rim of orbit).

***Pectoral fin*** moderately notched, with 19 (17–20) rays; upper lobe with 14 (12–15) rays, extending beyond (or just reaching) anal-fin origin; lower lobe elongate, with 5 (3–7) rays, uppermost ray of lower lobe longest, extending beyond anus, not reaching (reaching) anal-fin origin. Uppermost pectoral-fin base below a horizontal through posterior margin of maxillary. Lowermost pectoral-fin base below anterior rim of orbit (or below midway between tip of snout and anterior rim of orbit). Rays between upper and lower lobes widely spaced.

***Selected osteological characters*.** Roof of cranium comprising frontal and supraoccipital incompletely closed; frontal and supraoccipital poorly ossified; parietal absent (Fig. 2). Opercle well ossified, sharpened posteriorly, supporting upper margin of opercular flap. Subopercle thin, comprising two spines forming a V-shape; lower spine supporting lower margin of opercular flap. Subopercle and interopercle separated. Dorsal portion of cleithrum elongated. Proximal pectoral radials 4, rounded, moderately large and located medially (Fig. 3C). No interradial fenestrae between proximal radials. Scapula with strong helve, posterior margin with a small slit. Coracoid triangular with broad lamina. Distal radials present at base of all pectoral-fin rays, except for uppermost and lowermost rays.

***Coloration*.** In fresh specimens, head and body pale pink with fine melanophores; margin of preopercle silvery; anteroventral portion silvery, with dark peritoneum visible through thin skin; dorsal and anal fins crimson, distal margins somewhat darker; pectoral fin crimson (Fig. 1A). In preserved specimens, head, body, and fins pale with fine melanophores, somewhat larger posteriorly on body; peritoneum black, stomach dark brown (or black); orobranchial cavity pale with scattered melanophores.

***Reproductive characters*.** Ovary pouch-like, whitish; one of two ovaries with 33 ripe ovarian eggs (2.06–2.12 mm in diameter) and numerous unripe ovarian eggs (0.6 mm in maximum diameter) in female paratype (FAKU 147147, 80.4 mm SL). Testes slender, whitish (FAKU 147161, 51.5 mm SL).

#### Etymology.

The specific epithet *flammeus* is from Latin, meaning “flame”, and refers to the crimson fin coloration of the species.

#### Geographical distribution.

**Figure 4.**
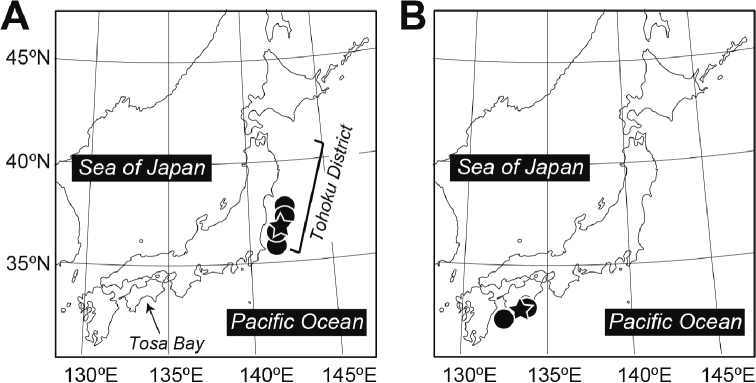
Distribution of specimens of (**A**) *Paraliparis flammeus* sp. nov. and (**B**) *P. mandibularis* examined during this study. Stars indicate type localities.

Western Pacific Ocean, off the Pacific coast of Tohoku District, northern Honshu, Japan, in depths of 422–890 m (Fig. 4A).

#### Remarks.

**Figure 5.**
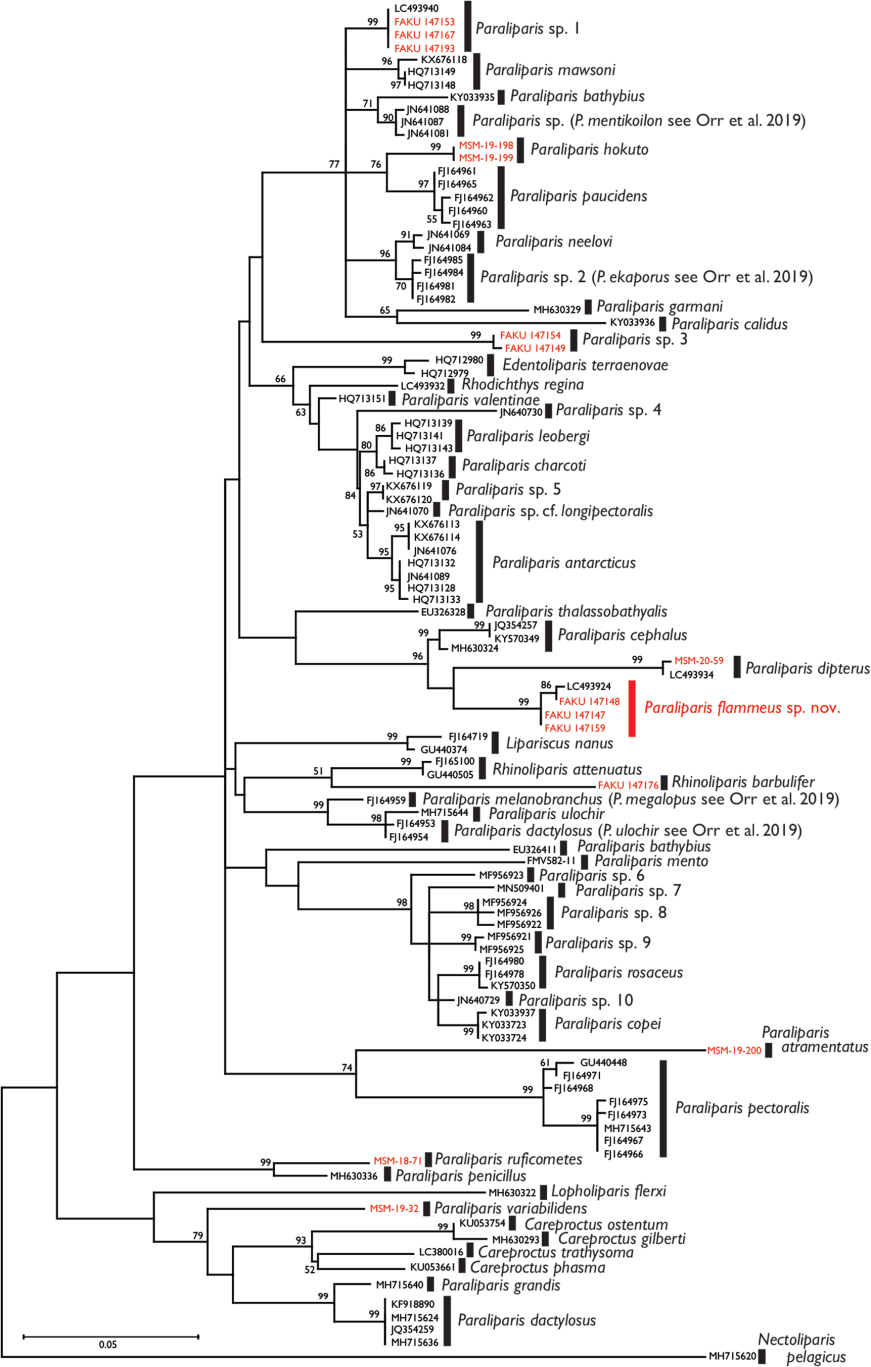
Maximum likelihood phylogenetic tree of *Paraliparis* and related genera based on COI sequences (492 bp). Support values (≥ 50% ML bootstrap probability) indicated along branches. Each node labeled with a registration number (red, determined in this study) or an accession number deposited in INSDC or BOLD. *Nectoliparis pelagicus* included as out-group.

Alignment of the COI gene sequences (492 bp) determined herein with previously determined sequences of *Paraliparis* resulted in a maximum likelihood tree based on 101 aligned sequences and the recovery of a monophyletic group comprising *P. flammeus*, *P. cephalus*, and *P. dipterus* (Fig. 5). Monophyly of the above species was supported by high bootstrap probability (95%). The uncorrected *p*-distance within *P. flammeus* was less than 0.006, strongly contrasting with values ranging from 0.030 to 0.067 for the above two species. *Paraliparis cephalus* is similar to *P. flammeus* in having an oblique mouth, but has 4 caudal-fin rays (vs 6 rays) and the uppermost pectoral-fin base above a horizontal through the maxillary posterior margin (Stein 1978; this study). *Paraliparis dipterus*, known only from the holotype collected from Suruga Bay, Japan, until the recent description of a developmental series by Takami and Fukui (2012), differs from *P. flammeus* in having a horizontal mouth, 12–14 pectoral-fin rays, and lacking pyloric caeca (present in *P. flammeus*) (Kido 1988; Takami and Fukui 2012; this study). The position of *P. mandibularis* is unknown due to the unavailability of sequence data.

Among the 28 species of *Paraliparis* known from the North Pacific, *P. flammeus* shares the morphological characters, i.e., an oblique mouth and the uppermost pectoral-fin base below a horizontal through the posterior margin of the maxillary, with only *P. mento* (Washington southward to Monterey Bay), *P. mandibularis* (Tosa Bay, Japan), and *Paraliparis angustifrons* (Garman, 1899) (off Panama) (Garman 1899; Kido 1988; Mecklenburg et al. 2002; Love et al. 2005; Baldwin and Orr 2010; Nakabo and Kai 2013; Murasaki et al. 2018, 2019a, b). However, *P. flammeus* differs from *P. mento* in having 6 caudal-fin rays (vs 5 rays) and greater preanal length (29.9–35.3% SL vs 26.7–28.5% SL), and from *P. mandibularis* in having 17–20 pectoral-fin rays (vs 27–30 rays) and a longer pectoral-fin lower lobe (16.7–23.4% SL vs. 13.8–15.9% SL). In addition, *P. angustifrons*, known only from two syntypes collected off Panama, also has an oblique mouth and low pectoral-fin position, but clearly differs from the new species in pectoral-fin ray number (37 in *P. angustifrons*) (Garman 1899; Chernova et al. 2004). Although *Paraliparis deani* Burke, 1912 (western Gulf of Alaska to northern California) and *Paraliparis melanobranchus* Gilbert & Burke, 1912 (southern Sea of Okhotsk and off British Columbia) are also similar to *P. flammeus* in the counts of dorsal- (56–57 and 52–60, respectively) and anal-fin rays (44–48 and 48–54, respectively), *P. flammeus* is distinguishable from *P. deani* in having a small gill slit entirely above the pectoral fin or extending ventrally to level of 1 or 2 uppermost pectoral-fin rays (vs extending to 10–13 pectoral-fin rays) and from *P. melanobranchus* in having 6 caudal-fin rays (vs 4) (Mecklenburg et al. 2002; Nakabo and Kai 2013). Among South Pacific species, *P. flammeus* resembles *Paraliparis membranaceus* Günther, 1877 and *Paraliparis molinai* Stein, Meléndez & Kong, 1991 in having an oblique mouth and low pectoral-fin position, but is distinguishable from both species by its lower pectoral-fin ray number (ca 25 in *P. membranaceus* and 24 in *P. molinai*) (Stein et al. 1991; Stein 2005).

### Paraliparis mandibularis

Taxon classificationAnimaliaScorpaeniformesLiparidae

Kido, 1985

894DC248-3008-58D6-BD30-7AEF22B020D2

**Figure 6.**
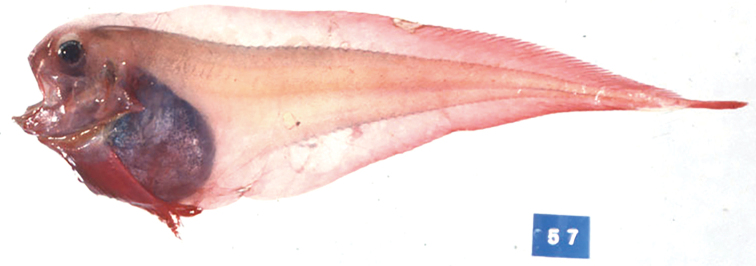
*Paraliparis mandibularis*, BSKU 43451, 128.1 mm SL, fresh specimen. Photo by BSKU.

Figs 3B, D , 6 [Japanese name: Ago-inkiuo]

Paraliparis mandibularis Kido 1985: 362, figs 2–4, Tosa Bay, Kochi, Japan; Kido 1988: 234, fig. 57 (modified from Kido 1985); Shinohara et al. 2001: 320, listed, Tosa Bay, Kochi, Japan; Nakabo and Kai 2013: 1217, key, unnumbered fig., Tosa Bay, Kochi, Japan.

#### Materials examined.

BSKU 30513 (holotype of *P. mandibularis*), 103.6 mm SL, 32.967°N, 133.533°E, 605 m depth, Tosa Bay, Kochi, Japan; BSKU 43451, 128.1 mm SL, 32.545°N, 132.433°E, 1,075–1,092 m depth, off Bungo Channel, Ehime, Japan; BSKU 44260, 44262, 44267, 44269 (C&S), 44397, 44398, 111.4–119.1 mm SL, 600 m depth, Tosa Bay, Kochi, Japan.

#### Diagnosis.

*Paraliparis mandibularis* is distinguished from other species of *Paraliparis* by the following combination of characters: mouth oblique; uppermost pectoral-fin base below a horizontal through posterior margin of maxillary; 63–66 vertebrae, 58–61 dorsal-fin rays, 52–54 anal-fin rays, 6 principal caudal-fin rays, and 27–30 pectoral-fin rays. Proximal pectoral radials 4, enlarged and moved to anterior edge of basal lamina. Parietals present. Among North Pacific species, it is similar to *P. flammeus* sp. nov., which differs from the former in having 17–20 pectoral-fin rays, and to *P. mento*, which has 5 principal caudal-fin rays.

#### Description.

Measurements are shown in Table 1. Body compressed, elongate, deepest at nape, taping posteriorly (Fig. 5). Skin thin, fragile. Head compressed, dorsal profile strongly sloping from nape to snout. Snout deep, abruptly angled, its length almost equal to orbit diameter; not projecting anterior to upper jaw. Mouth strongly oblique, lower jaw slightly protruding beyond (or almost same length as) upper jaw; premaxillary tooth plates matching mandibular tooth plates; maxilla extending to posterior margin of orbit; oral cleft extending to middle of orbit. Premaxillary teeth simple, in 3–6 oblique rows; diastema narrow between premaxillae. Mandibular teeth simple, in 4 or 5 oblique rows (Fig. 3B); diastema absent at lower jaw symphysis. Orbit of moderate size, rounded. Nostril single, without distinct tube, slightly above level of mid-orbit. Cephalic sensory pores small: nasal pores 2, maxillary pores 6, preoperculomandibular pores 7, suprabranchial pore 1; cephalic pore pattern 2-6-7-1. Chin pores paired, openings well separated on skin surface. Coronal pore absent. Gill slit moderately large, upper margin level with mid-orbit, extending ventrally to just above pectoral fin or to level of 1–3 uppermost pectoral-fin rays. Gill rakers 10–12, blunt and small. Tip of opercular flap sharp, directed posteriorly, level with mid-orbit or posterior margin of maxillary.

**Table 1.** Measurements of *Paraliparis flammeus* sp. nov. and *P. mandibularis* (means in parentheses).

	***Paraliparis flammeus***		***Paraliparis mandibularis***	
	**Holotype**	**Paratypes**	**Holotype**	**Non-types**
	**MSM-20-52**	***n* = 14**	**BSKU 30513**	***n* = 6**
Standard length (mm)	75.8	42.5–80.4	103.6	104.7–128.1
In % of standard length				
Head length	21.4	17.2–24.1 (20.8)	19.8	18.0–20.7 (19.8)
Snout length	6.7	5.2–7.0 (6.2)	6.9	5.6–6.9 (6.1)
Orbit length	5.8	4.5–6.2 (5.4)	5.6	4.4–6.0 (5.2)
Interorbital width	7.2	3.6–8.8 (5.8)	8.0	5.9–7.7 (6.5)
Maxilla length	10.8	10.6–12.0 (11.1)	10.5	9.7–11.1 (10.6)
Gill slit length	10.3	6.4–10.9 (8.4)	6.9	7.5–10.5 (9.1)
Body depth	23.0	13.5–21.7 (18.6)	16.1	16.0–24.9 (19.8)
Pectoral-fin length	26.2	18.5–26.7 (22.4)	Damaged	21.6–26.0 (23.3)
Pectoral-fin lower lobe length	19.6	16.7–23.4 (20.6)	Damaged	13.8–15.9 (15.1)
Pectoral-fin notch-ray length	13.3	9.1–15.9 (12.6)	Damaged	9.6–11.8 (10.7)
Predorsal length	22.4	20.3–24.5 (22.5)	18.5	19.7–23.7 (21.2)
Preanal length	35.3	29.9–35.2 (29.9)	33.2	31.8–36.1 (33.8)
Snout to anus length	16.9	13.0–16.7 (14.5)	13.2	12.0–18.8 (14.6)
Caudal-fin length	11.8	10.8–15.1 (12.9)	Damaged	15.5–19.6 (17.8)
In % of head length				
Snout length	31.3	24.5–35.5 (29.9)	34.6	27.4–34.2 (30.9)
Orbit length	27.1	21.7–32.3 (26.1)	28.2	23.2–29.0 (26.5)
Interorbital width	33.8	20.6–36.6 (27.8)	40.5	29.8–35.1 (33.0)
Maxilla length	50.5	43.8–63.7 (53.7)	52.8	51.8–56.3 (53.5)
Gill slit length	48.1	30.5–18.3 (40.5)	34.7	37.4–51.9 (46.6)
In % of caudal-fin length				
Dorsal-fin connection to caudal fin	37.9	22.3–37.7 (29.6)	Damaged	38.4–50.6 (42.9)
Anal-fin connection to caudal fin	40.1	29.2–44.7 (36.9)	Damaged	19.6–36.4 (29.8)

***Dorsal-fin*** rays 58–63; anteriormost ray above tip of opercle, posteriormost ray attached membranously to dorsalmost caudal-fin ray. Anteriormost dorsal-fin pterygiophore inserted between neural spines 3 and 4 or 4 and 5, bearing a single ray. Anal-fin rays 52–54; posteriormost ray attached membranously to ventralmost caudal-fin ray. Vertebrae 63–66, comprising precaudal 9 and caudal 54–57. Pleural ribs absent. Hypurals and parhypural fused into single plate. Caudal fin slender, posterior margin slightly rounded. Principal caudal-fin rays 6, dorsal principal rays 3, ventral principal rays 3, no procurrent rays. Pyloric caeca 5 or 6, short and finger-like, on left side of visceral cavity. Anus below posterior margin of orbit.

***Pectoral fin*** moderately notched, with 27–30 rays; upper lobe with 17–19 rays, extending beyond (or just reaching) anal-fin origin; lower lobe elongate, with 8–13 rays, uppermost ray of lower lobe longest, extending beyond anus, not reaching anal-fin origin. Uppermost pectoral-fin base below a horizontal through posterior margin of maxillary. Lowermost pectoral-fin base below anterior rim of orbit or below midway between tip of snout and anterior rim of orbit. Rays between upper and lower lobes widely spaced.

***Selected osteological characters*.** Roof of cranium without distinct crest comprising well ossified frontals, supraoccipital, and parietals. Opercle well ossified, sharpened posteriorly, supporting upper margin of opercular flap. Subopercle thin, comprising two spines forming a V-shape; lower spine supporting lower margin of opercular flap. Subopercle and interopercle attached. Cleithrum broad and robust, dorsal portion elongated. Proximal pectoral radials 4, enlarged occupying almost entire width of cartilaginous basal laminae and moved to anterior edge of basal lamina (Fig. 3D). No interradial fenestrae between proximal radials. Scapula with strong helve. Coracoid narrowly triangular with narrow lamina. Distal radials absent.

***Coloration*.** In fresh specimens, head and body pale pink, posterior half of body reddish; dark peritoneum visible through thin skin; dorsal and anal fins pale pink, distally reddish; caudal and pectoral fins red (Fig. 6). In preserved specimens, head, body, and fins pale; peritoneum black, stomach pale or white; orobranchial cavity pale with scattered melanophores.

#### Geographical distribution.

Western Pacific Ocean; Tosa Bay and Bungo channel, off Shikoku Island, Japan, in depths of 600–1,092 m (Kido 1988; this study).

#### Remarks.

*Paraliparis mandibularis* was originally described by Kido (1985) on the basis of a single specimen collected from Tosa Bay, Japan. Subsequently, Kido (1988) redescribed the species on the basis of the holotype and an additional non-type specimen. However, a number of details, including osteology and fresh coloration have remained unknown to date. Whereas the pectoral girdle in *Paraliparis* species generally has a reduced number and size of radials (Andriashev 1998; Orr et al. 2019), that of *P. mandibularis* has four broad robust radials (Fig. 3D). The species is also characterized by a pair of parietals in the cranium, such being absent in some other species of *Paraliparis* (Kido 1988). Although Kido (1988) described *P. mandibularis* as having a diastema at the symphysis of both the upper and lower jaws, we could find no obvious diastema at the lower jaw symphysis in the specimens examined here, including the holotype. In addition to the differences described above between *P. mandibularis* and *P. flammeus*, the former is further distinguished from the latter by the enlarged pectoral radials occupying almost entire width of the cartilaginous basal laminae and moved to the anterior edge of basal lamina in the pectoral girdle (vs moderately large and medial) and the parietals in the cranium (vs. absent). *Paraliparis mandibularis* differs from other species with an oblique mouth and a low positioned pectoral fin, viz. *P. angustifrons*, *P. membranaceus*, and *P. molinai* (see Remarks under *P. flammeus*), in having 27–30 pectoral-fin rays (vs 37 in *P. angustifrons*, ca 25 in *P. membranaceus*, 24 in *P. molinai*). *Paraliparis membranaceus* and *P. molinai* have similar pectoral-fin ray numbers to *P. mandibularis* but have a reduced caudal fin (4 rays in *P. molinai*, 2 or 3 in *P. membranaceus* vs 6 in *P. mandibularis*) (Stein 2005).

## Discussion

Jordan and Evermann (1896) designated the subgenus Amitrichthys under *Paraliparis*, the former including *P. mento*, *P. cephalus*, *Paraliparis rosaceus* Gilbert, 1890, *Paraliparis copei* Goode & Bean, 1896, and *Paraliparis dactylosus* Gilbert, 1896. Recently, however, Orr et al. (2019) included on the basis of their molecular phylogenetic tree only *P. mento* and *P. cephalus* in *Amitrichthys*. In the molecular phylogenetic tree presented herein, *P. flammeus* has a monophyletic relationship with *P. cephalus* and *P. dipterus*, whereas *P. mento* is included in a clade comprising *P. rosaceus*, *P. copei*, and some other, undescribed species (Fig. 5). Because the positions of *P. mandibularis*, *P. angustifrons*, *P. membranaceus*, and *P. molinai* are still unknown due to a lack of available COI sequences, the bootstrap probabilities supporting each node of both the current phylogenetic tree and that of Orr et al. (2019) are generally low, and the significant difference in osteological characters between *P. flammeus* and *P. mandibularis* may be indicative of a distant relationship, we remain conservative in avoiding a redefinition of the subgenus Amitrichthys at present. Further studies are required, based on longer sequences and comprehensive taxon sampling, for clarification of the phylogenetic relationships within *Paraliparis*.

*Paraliparis flammeus* is characterized by a poorly ossified cranium, a characteristic recognized elsewhere in some snailfishes, including *Nectoliparis pelagicus*, *Pseudoliparis swirei* Gerringer & Linley, 2017 and *Rodichthys regina* Collett, 1879 (Kido 1988; Wang et al. 2019). Although Wang et al. (2019) reported a genetic change associated with adaptation to the deep sea in *P. swirei* (known only from hadal depths in the Mariana Trench, 6,198–8,098 m; Gerringer et al. 2017) as resulting in reduced cranial structure, *P. flammeus* is known from 422–890 m, *N. pelagicus* from ~549 m (Love et al. 2005), and *R. regina* from 400–2,365 m (Chernova et al. 2004; Mecklenburg et al. 2018), suggesting that incomplete cranial structure is not necessarily a result of adaptation for hadal depths. It should be noted that, unlike demersal *P. swirei*, characterized by a large pelvic disk (Gerringer et al. 2017), *P. flammeus*, *N. pelagicus*, and *R. regina* lack a pelvic disk and are apparently mesopelagic (Mecklenburg et al. 2002, 2018). Similarly, a mesopelagic genus of bobtail snipe eel, *Neocyema* Castle, 1978, is known to have an incompletely ossified cranium, possibly suggesting neoteny or a trade-off between reduction of some bony structures (Poulsen 2015).

## Comparative material

*Paraliparis atramentatus*: MSM-19-200 (INSDC accession: LC556302), 38.8 mm SL, 35.003°N, 138.543°E, Suruga Bay, Japan, 253–1,282 m depth; *Paraliparis cephalus*: USNM 325577 (2 specimens), 76.7 mm SL, SL unknown, 59.328°N, 178.112°W, 603–610 m depth, Bering Sea; UW 153315, 30.2+ mm SL, 39.980°N, 124.675°W, 1,088 m depth. *Paraliparis dipterus*: MSM-20-58, 37.8 mm SL, MSM-20-59 (LC556303), 35.7 mm SL, 35.002°N, 138.548°E, 501–935 m depth, Suruga Bay, Japan. *Paraliparis hokuto*: MSM-19-198, holotype (LC556304), 192.7 mm SL, 34.982°N, 138.632°E, 1,432–1,554 m depth, Suruga Bay, Japan; MSM-19-199, paratype (LC556305), 138.3 mm SL, 34.985°N, 138.648°E, 1,462–1,562 m depth, Suruga Bay, Japan. *Paraliparis mento*: UW 115470, 99.3 mm SL, 44.928°N, 124.979°W, 800 m depth; UW 150606, 85.6 mm SL, 44.352°N, 124.856°W, 822 m depth; UW 151861, 83.4 mm SL, 41.463°N, 124.567°W. *Paraliparis ruficometes*: MSM-18-71, paratype (LC556301), 65.7 mm SL, 34.981°N, 138.632°E, 1,430–1,560 m depth. *Paraliparis variabilidens*: MSM-19-32, holotype (LC556300), 55.9 mm SL, 34.985°N, 138.648°E, Suruga Bay, Japan, 1,462–1,562 m depth; *Paraliparis* sp. 1 (see Fig. 5): FAKU 147153 (LC556306), 92.9 mm SL, 36.807°N, 141.613°E, 752 m depth; FAKU 147167 (LC556307), 62.8 mm SL, 36.523°N, 141.203°E, 653 m depth; FAKU 147193 (LC556308), 70.4 mm SL, off Tohoku, Japan. *Paraliparis* sp. 3 (see Fig. 5): FAKU 147149 (LC556309), 57.1 mm SL, 36.842°N, 141.578°E, 647 m depth; FAKU 147154 (LC556310), 69.4 mm SL, 36.807°N, 141.613°E, 752 m depth. *Rhinoliparis barbulifer*: FAKU 147176 (LC556314), 38.346°N, 142.100°E, 485 m depth.

## Supplementary Material

XML Treatment for Paraliparis flammeus

XML Treatment for Paraliparis mandibularis
